# Sensory processing atypicalities and social responsiveness in autism spectrum disorder: the mediation of executive function

**DOI:** 10.3389/fpsyt.2025.1696983

**Published:** 2026-01-20

**Authors:** Yue Ji, Feng-lei Zhu, Pei-pei Yin, Shu-ting Zeng, Zhi Huang

**Affiliations:** Child Developmental-Behavioral Center, The Third Affiliated Hospital of Sun Yat-sen University, Guangzhou, China

**Keywords:** autism spectrum disorders, children, executive function, mediation, sensory processing, social responsiveness

## Abstract

**Background:**

Individuals with autism spectrum disorder (ASD) commonly display challenges in social interaction, executive functioning, and sensory processing. Nevertheless, the interrelationships among these domains are not yet fully understood. This study aimed to elucidate whether sensory processing affects social functioning in children with ASD through the mediating role of executive functioning.

**Methods:**

A total of 88 children and adolescents with ASD, aged 7 to 14 years, were enrolled. Parent-reported measures included the social responsiveness scale (SRS; social functioning), the behavior rating inventory of executive function–second edition (BRIEF-2; executive functioning), and the sensory profile–second edition (SP-2; sensory processing). Mediation analysis was conducted to examine the proposed relationships.

**Results:**

The results indicated that emotional regulation, a component of executive functioning, fully mediated the relationship between sensory avoiding and social responsiveness, whereas it partially mediated the relationship between sensory registration and social responsiveness. Additionally, behavioral regulation, another dimension of executive functioning, partially mediated the effects of both sensory avoiding and sensory registration on social responsiveness. These relationships remained significant after controlling for gender, age, and intelligence.

**Conclusion:**

These findings underscore the importance of both sensory processing and executive functioning in the social responsiveness of children with ASD. The results suggest a potential mechanistic framework in which executive functions serve as a mediating factor between sensory processing and social behavior in this population.

## 1 Introduction

Autism spectrum disorder (ASD) is one of the most prevalent neurodevelopmental disorders of childhood (1), characterized by core impairments in social communication as well as restricted interests and repetitive behaviors (2). Atypical sensory processing—affecting an estimated 45% to 95% of individuals with ASD (3–5), likely due to differences in measurement tools, sample characteristics, and diagnostic criteria—is increasingly recognized as a defining feature of the condition (6). The diagnostic and statistical manual of mental disorders, fifth edition (DSM-5) acknowledges sensory anomalies as part of the behavioral manifestations associated with repetitive behaviors (2, 7). These sensory atypicalities span multiple domains, including visual, auditory, gustatory, olfactory, proprioceptive, vestibular, and tactile systems, with evidence suggesting that nearly all primary sensory modalities are affected in ASD (4, 6, 8–10). Beyond their diagnostic significance, sensory processing differences are associated with deficits in social cognition, executive functioning, and the severity of other core ASD symptoms (3, 11, 12). For instance, Stevenson et al. (2018) reported that deficits in audiovisual integration exacerbate difficulties in speech processing and social information synthesis (13), while Green et al. (2018), using fMRI, demonstrated that heightened sensory responsivity can divert attentional resources away from social stimuli (14). Sensory processing challenges are also linked to emotional dysregulation, wherein elevated sensory input may provoke anxiety (15). Furthermore, Grinblat et al. (2019) proposed that atypical sensory processing may overwhelm attentional resources, thereby constraining the effective deployment of executive functions such as task initiation and attentional shifting (16). Jessica M et al. (2019) reported that (17), compared with typically developing peers, adolescents with ASD show heightened autonomic responses to noise—evidenced by increased heart rate and elevated skin conductance—when performing demanding tasks. This elevated arousal may, in turn, impair performance on complex cognitive tasks. According to the Yerkes–Dodson law (18), task performance is optimized at moderate arousal levels, whereas both insufficient and excessive arousal reduce performance. Atypical sensory processing in ASD may therefore shift individuals away from this optimal arousal window, ultimately affecting higher-order cognitive functioning.

In addition to core and sensory features, children with ASD commonly exhibit impairments in executive functions (EFs) (19–21). Executive functions comprise higher-order cognitive control processes essential for goal-directed behavior, including planning, working memory, inhibitory control, cognitive flexibility, and task monitoring (22, 23). A systematic review by Demetriou et al. (2020) concluded that EF impairments are pervasive in ASD, though characterized by considerable individual variability (24). Marciszko et al. (2020) further indicated that EF deficits are associated with reduced social competence, suggesting a mechanistic link between cognitive regulation and social functioning (25, 26). Both sensory processing and executive functions are considered foundational capacities supporting the development of more complex social communication skills (12, 25, 27, 28).

Growing evidence suggests that sensory processing, executive functioning, and social functioning are interrelated in ASD, with early sensory differences potentially disrupting higher-order cognitive processes (29). Executive functioning may thus serve as a key mechanism translating sensory disruptions into social difficulties. Lane et al. (2009), for example, observed significant associations between executive functioning, sensory atypicalities, and communication challenges (30). Although understanding the mediating role of executive functioning in the sensory–social relationship is crucial, empirical evidence remains limited. Fernandez-Prieto et al. (2021) found that emotional control—a subdomain of EF—mediated the relationship between sensory processing and behavioral outcomes in children and adolescents with ASD (31). However, their study focused specifically on emotional regulation, inhibitory control, and working memory, rather than examining the full range of executive functions.

Therefore, the present study proposes a mediation model hypothesizing interrelationships among sensory processing, executive functioning, and social responsiveness in ASD, positing that sensory processing influences social responsiveness both directly and indirectly through executive functioning. By investigating the dynamic interplay among these domains in children with ASD, this research aims to provide a novel theoretical framework for understanding how sensory processing affects social functioning, while also offering an empirical basis for developing targeted clinical interventions.

## 2 Materials and methods

### 2.1 Participants

A total of 88 children diagnosed with ASD were enrolled in this study. Participants were primarily recruited through online advertisements targeting families in southern China, with additional referrals from the child development and behavioral center at the third affiliated hospital of Sun Yat-sen university. The study was approved by the ethics committee of the third affiliated hospital of Sun Yat-sen university, and written informed consent was obtained from all participants and their legal guardians.

Eligibility was initially determined through an online screening interview. Inclusion criteria for the ASD group were: (1) age between 7 and 14 years; (2) a full-scale intelligence quotient (FSIQ) > 70 as assessed by the Wechsler intelligence scale for children–fourth edition (WISC-IV); and (3) meeting diagnostic criteria on both the autism diagnostic interview–revised (ADI-R) (32) and the autism diagnostic observation schedule–second edition (ADOS-2) (33). Final diagnosis was confirmed by a developmental-behavioral pediatrician according to DSM-5 criteria. Participants with a known genetic syndrome or FSIQ below 70 were excluded, in light of evidence suggesting atypical sensory response patterns in genetic disorders and the challenges inherent in assessing executive functions in children with intellectual disability (34).

### 2.2 Measures

#### 2.2.1 Sensory profile 2

The Chinese version of the sensory profile 2 (SP-2) was used to evaluate sensory processing patterns (35). This parent-report instrument consists of 86 items rated on a 5-point Likert scale and is designed for children aged 3–14 years. It assesses four sensory processing quadrants—seeking, avoiding, sensitivity, and registration—across six sensory domains. For this study, quadrant scores were analyzed, with higher scores indicating heightened sensory reactivity. Internal consistency was high across quadrants (Cronbach’s α = 0.847–0.896), comparable to the reliability reported for the original English version (0.60–0.90) (36). The Chinese SP-2 has been increasingly used in sensory assessments of children in China (37, 38).

#### 2.2.2 Behavior rating inventory of executive function, second edition

Executive functioning was assessed using the parent-report form of the Chinese BRIEF-2, which contains 63 items evaluating various aspects of executive function in children aged 5–18 years (39–41). The measure yields three composite indices—behavioral regulation index (BRI), emotional regulation index (ERI), and cognitive regulation index (CRI)—and a global executive composite (GEC). Higher scores indicate greater executive dysfunction, and positive correlations with SRS scores reflect more pronounced deficits. This study focused on the three composite indices. The Chinese BRIEF-2 has demonstrated good reliability and has been increasingly used in children in China (39–41). Reported internal consistency coefficients ranging from 0.66 to 0.95 (39), and in the present sample, internal consistency was 0.83–0.924 for the composite indices and 0.614–0.824 for the subscales, comparable to the original version (42).

#### 2.2.3 Social responsiveness scale

The SRS is a 65-item rating scale designed to quantify social impairments (43) and assess autism-like symptoms exhibited in natural settings over the past six months. The items are organized into five subdomains—social awareness, social cognition, social communication, social motivation, and restricted and repetitive behaviors. Each item is rated on a 4-point Likert scale, with higher scores indicating more pronounced ASD-related traits. SRS scores show strong correlations with the gold-standard diagnostic measure, the ADI-R (44). The Chinese version demonstrates excellent reliability and validity (45), with Cronbach’s α coefficients ranging from 0.94 to 0.95. It has been widely used in assessments of autistic traits and symptoms among children in China (46, 47). The present study employed the parent-report version of the Chinese SRS.

#### 2.2.4 Wechsler intelligence scale for children, fourth edition

The Wechsler intelligence scale for children, fourth edition (WISC-IV) (48), consists of 10 core subtests and 5 supplemental subtests. The core subtests are used to derive four factor indices. The verbal comprehension index (VC) is based on the similarities, vocabulary, and comprehension subtests. The perceptual reasoning index (PR) is based on block design, matrix reasoning, and picture concepts. The working memory index (WM) is based on digit span and letter–number sequencing. The processing speed index (PS) is based on coding and symbol search. The full-scale IQ (FSIQ) is derived from the 10 core subtests and used as an indicator of intellectual functioning.

### 2.3 Statistical analysis

All analyses were conducted using SPSS 25.0 and the PROCESS (v4.2) for mediation modeling (49). Normality assumptions were verified using the Shapiro–Wilk test, and no transformations were necessary. Mediation analyses were performed to examine whether executive functioning mediates the relationship between sensory processing and social responsiveness, controlling for sex, age, and FSIQ. Separate models were tested for each sensory quadrant (avoiding, registration, sensitivity, seeking) and executive function composite (BRI, ERI, CRI). Significant indirect effects (a×b paths) were tested using bias-corrected bootstrapping with 5,000 resamples. For each model, standardized beta weights (β), standard error (SE), and 95% confidence intervals (CI) are reported. Although the measures assess overlapping constructs such as regulation and attention, the mediation models were constructed to test specific theoretical pathways rather than conflate domains. Only sensory avoiding and registration showed significant mediation effects and are reported herein. A sensitivity analysis conducted in G*Power 3.1 (50) indicated that a sample size of 64 would provide 80% power to detect an effect size of R² = 0.16 at α = 0.05 with two predictors.

For all instruments, standardized scores were used in analyses to facilitate interpretation and comparison. Specifically, the SRS and BRIEF-2 provided T-scores, while the SP-2 quadrant scores were reported as raw scores, as these are commonly used in clinical and research contexts to reflect the frequency and severity of sensory processing behaviors. The use of parent-reported measures was chosen for their ecological validity and ability to capture behaviors across multiple contexts. However, this approach may be subject to reporting biases, such as social desirability or subjective interpretation. To mitigate this, future studies could incorporate behavioral tasks or observational measures to complement parent reports.

## 3 Results

### 3.1 Descriptive statistics

Descriptive statistics for all study variables, including demographic characteristics, social responsiveness, sensory processing profiles, and executive functioning indices, are summarized in **Table 1**. The sample consisted of 88 children with ASD (76 males, 12 females) with a mean age of 10.04 years (SD = 2.07). The mean FSIQ was 94.30 (SD = 15.80). Clinical interpretation of the scores indicated that the mean SRS Total Score (84.92 ± 21.19) and BRIEF-2 indices (GEC: 128.20 ± 19.78) fell within clinically significant ranges, suggesting substantial social impairment and executive dysfunction in the sample.

**Table 1:** Demographic and clinical characteristics of participants with ASD.

Variables	ASD participants(N = 88)
Sex (male:female)	76:12
Age, years (M ± SD)	10.04 ± 2.07
FSIQ	94.30 ± 15.80
SRS	
SRS Total Score	84.92 ± 21.19
Social Awareness	11.24 ± 3.10
Social Cognition	17.28 ± 4.11
Social Communication	28.28 ± 8.41
Social Motivation	12.58 ± 4.69
Restricted/Repetitive Behaviors	15.53 ± 6.10
SP-2 Quadrants	
Seeking	43.77 ± 12.06
Avoiding	54.36 ± 12.06
Sensitivity	45.03 ± 11.21
Registration	55.15 ± 14.87
BRIEF-2 Indices	
Global Executive Composite (GEC)	128.20 ± 19.78
Behavioral Regulation Index (BRI)	26.78 ± 4.53
Emotional Regulation Index (ERI)	29.37 ± 6.08
Cognitive Regulation Index (CRI)	72.05 ± 11.93

ASD, Autism spectrum disorder; M ± SD, Mean ± standard deviation; FSIQ, Full-scale intelligence quotient; SRS, Social responsiveness scale; SP-2, Sensory profile-2; BRIEF-2, Behavior rating inventory of executive function–second edition.

### 3.2 Correlational analyses

As presented in **Table 2**, significant correlations were observed among sensory processing patterns, executive functioning, and social responsiveness. The SRS total score was significantly correlated with all four sensory processing quadrants. Each sensory processing dimension was also significantly associated with all three composite indices of the BRIEF-2. Similarly, social responsiveness showed significant positive correlations with the behavioral regulation index (BRI), emotional regulation index (ERI), and cognitive regulation index (CRI).

**Table 2:** Pearson correlations among sensory processing, executive function, and social responsiveness.

Variables	Seeking	Avoiding	Sensitivity	Registration	SRS total
SRS total	0.251*	0.490***	0.303**	0.476***	1
BRI	0.522***	0.522***	0.522***	0.522***	0.595***
ERI	0.300**	0.679***	0.436***	0.488***	0.581***
CRI	0.433***	0.511***	0.576***	0.597***	0.382***

*p < 0.05, **p < 0.01, ***p < 0.001; SRS: Social responsiveness scale; BRI: Behavioral regulation index; ERI: Emotional regulation index; CRI: Cognitive regulation index.

### 3.3 Mediation analysis

Mediation analyses were conducted to examine whether executive function mediates the relationship between sensory processing and social responsiveness, controlling for sex, age, and FSIQ. Sensory sensitivity and seeking did not yield significant mediation effects (all p >0.05, 95% CIs included zero), and thus were excluded from further mediation reporting.

**Figure 1:**
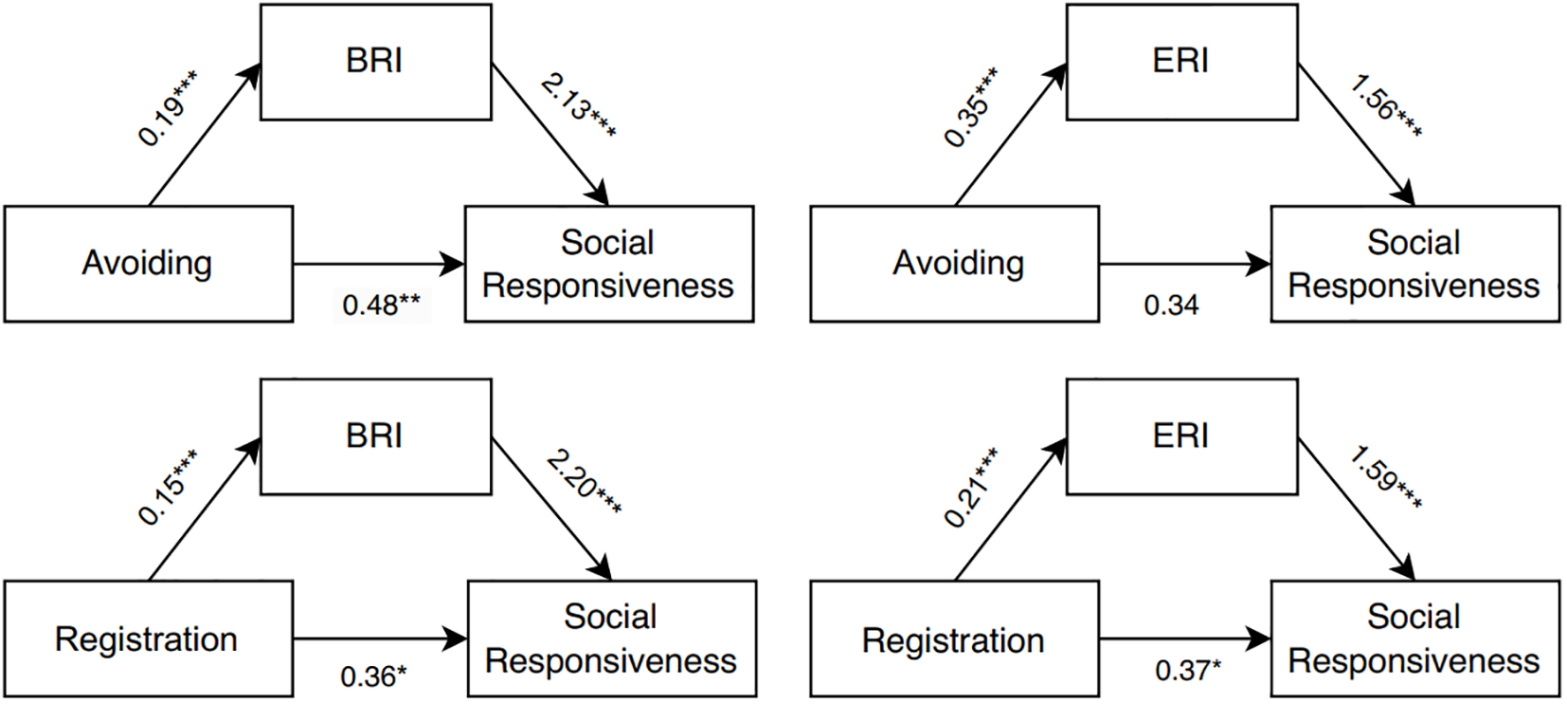
Mediation models illustrating the relationships among sensory processing, executive function, and social responsiveness in children with ASD. The top row shows mediation effects of BRI and ERI between Sensory Avoiding and Social Responsiveness. The bottom row shows mediation effects of BRI and ERI between Sensory Registration and Social Responsiveness; BRI, Behavioral Regulation Index; ERI, Emotional Regulation Index; ***p < 0.001, **p < 0.01, *p < 0.05.

The results, as summarized in **Table 3** and illustrated in **Figure 1**, indicated that both BRI and ERI significantly mediated the relationships between sensory processing patterns and social responsiveness. Specifically, the total effect of sensory avoiding on social responsiveness was significant (β = 0.89, SE = 0.17, p < 0.001). The indirect effect through BRI was significant (β = 0.41, SE = 0.12, 95% BCa CI [0.21, 0.67]), accounting for 46% of the total effect, indicating partial mediation. The indirect effect through ERI was also significant (β = 0.54, SE = 0.18, 95% BCa CI [0.20, 0.91]), accounting for 61% of the total effect, indicating full mediation as the direct effect became non-significant (β = 0.34, SE = 0.22, p = 0.112).

**Table 3:** Mediation analysis results for executive function mediating sensory-processing–social-responsiveness relationships.

Model (X → M → Y)	Path a	Path b	Direct effect (c’)	95% BCa CI	Effect size (ab/c)
X: Avoiding → Y: SRS					
BRI	0.19 ± 0.04***	2.13 ± 0.47***	0.48 ± 0.18**	[0.21, 0.67]	0.46
ERI	0.35 ± 0.04***	1.56 ± 0.42***	0.34 ± 0.22	[0.20, 0.91]	0.61
CRI	0.52 ± 0.09***	0.29 ± 0.20	0.74 ± 0.20***	[−0.05, 0.36]	0.17
X: Registration → Y: SRS					
BRI	0.15 ± 0.03***	2.20 ± 0.47***	0.36 ± 0.14*	[0.17, 0.55]	0.49
ERI	0.21 ± 0.04***	1.59 ± 0.35***	0.37 ± 0.14*	[0.15, 0.53]	0.47
CRI	0.50 ± 0.07***	0.25 ± 0.21	0.57 ± 0.18**	[−0.10, 0.34]	0.18

All models control for sex, age, and FSIQ. Unstandardized coefficients reported; ***p < 0.001, **p < 0.01, *p < 0.05; BCa CI, bias-corrected and accelerated confidence interval; BRI, Behavioral regulation index; ERI, Emotional regulation index; CRI: Cognitive regulation index.

Similarly, the total effect of sensory registration on social responsiveness was significant (*β* = 0.69, *SE* = 0.14, *p* < 0.001). The indirect effect through BRI was significant (*β* = 0.34, *SE* = 0.10, 95% *BCa CI* [0.17, 0.55]), accounting for 49% of the total effect, indicating partial mediation. The indirect effect through ERI was also significant (*β* = 0.33, *SE* = 0.10, 95% *BCa CI* [0.15, 0.53]), accounting for 47% of the total effect, indicating partial mediation. CRI did not show significant mediating effects in any model. ,

## 4 Discussion

This study examined the interrelationships among atypical sensory processing, executive function, and social responsiveness in children with ASD, with a specific focus on the mediating role of executive function between sensory processing abnormalities and social behavior. To our knowledge, this is the first study to incorporate all three core domains of executive function—behavioral, emotional, and cognitive regulation—into a unified mediation model linking sensory processing to social outcomes. The results supported our hypotheses: significant intercorrelations were observed among sensory processing, executive function, and social responsiveness, and executive function mediated the relationship between sensory atypicalities and social behavior, independent of age, sex, or FSIQ. These findings provide mechanistic insights into the sensory–executive–social pathway in ASD and highlight potential targets for intervention.

### 4.1 Associations among sensory processing, executive function, and social responsiveness

All four sensory processing dimensions—sensitivity, seeking, avoiding, and registration—were significantly correlated with behavioral, emotional, and cognitive regulation. This aligns with previous studies reporting that sensory hyper- or hypo-reactivity is associated with emotional dysregulation in children with ASD (51, 52). Kenworthy et al. (2009), for example, suggested that abnormal tactile responsivity and balance issues may contribute to impaired emotional regulation (53).

From a neurobiological perspective, sensory information is processed hierarchically, from primary sensory cortices to higher-order association areas that interact with prefrontal regions responsible for executive control (54). Aberrant sensory input may disrupt top-down regulatory mechanisms, contributing to executive dysfunction and subsequent social deficits. This aligns with models emphasizing the role of the salience network and fronto-insular circuits in integrating sensory and social information (55).

Furthermore, each sensory processing dimension was significantly associated with social responsiveness, corroborating earlier findings. Nada et al. (2019), for instance, reported that greater sensory abnormalities were linked to more severe social deficits as measured by the SRS-2 (56). Similarly, Thye et al. (2018) emphasized in a review that despite the heterogeneity of sensory profiles in ASD, atypical sensory processing consistently predicts social functioning (29). Extending these results, Crasta et al. (2024) identified registration difficulties as the strongest predictor of social behavior in adults with ASD, followed by avoiding and sensitivity (57).

### 4.2 Executive function and social responsiveness

Behavioral regulation—which includes inhibitory control and self-monitoring—plays a critical role in modulating social behavior. Deficits in this domain may impair social awareness, social cognition, communication, and motivation, thereby compromising overall social functioning. Among adolescents with ASD, impairments in behavioral regulation have been shown to predict deficits in language communication (58) and broader social interactions (59). Emotional regulation, encompassing cognitive flexibility and emotional control, facilitates adaptive responses to changing social contexts. Bertollo et al. (2020) found that impairments in shifting and emotional control were associated with social-communication challenges in children with ASD (60), while Brown et al. (2021) reported strong links between emotional dysregulation and both sensory avoidance and registration difficulties (61). Cognitive regulation—including initiation, working memory, planning, and organization—also influences social outcomes. Bednarz et al. (2020) demonstrated that metacognitive skills predict social awareness and communication in adolescents with ASD (62).

### 4.3 Executive function as a mediator between sensory processing and social responsiveness

As hypothesized, executive function significantly mediated the relationship between sensory processing and social responsiveness. Specifically, behavioral and emotional regulation mediated the effects of sensory avoiding and registration on social outcomes. Behavioral regulation partially mediated the relationships between both sensory avoiding and registration and social responsiveness, while emotional regulation showed a full mediation effect in the statistical model, though this should be interpreted cautiously given the cross-sectional nature of the data and does not imply the absence of other causal pathways. In contrast, cognitive regulation did not exhibit a significant mediating effect, suggesting that behavioral and emotional regulatory processes are more directly involved in translating sensory atypicalities into social challenges.

These findings extend previous work by Fernandez-Prieto et al. (2021), who identified emotional control as a mediator between sensory processing and social outcomes (31). Our results not only confirm the role of emotional regulation but also reveal a novel mediating contribution of behavioral regulation. This is consistent with models proposing that effective behavioral adaptation requires the integration of external sensory information with internal standards such as prior experience and motivational states (55).

Children with registration difficulties may fail to detect relevant sensory cues, impairing their ability to respond adaptively in social situations. Deficits in behavioral regulation can exacerbate these issues, supporting the finding of Gianluca et al. (2016) regarding associations among registration difficulties, emotion recognition deficits, and impulsivity (63). Conversely, children with sensory avoiding behaviors—often characterized by low sensory thresholds—may experience sensory overload, hyperarousal, and anxiety (51), which disrupt emotional regulation and amplify social deficits (64). Excessive attention to sensory stimuli may also impair the filtering and prioritization necessary for executive control, further compromising social functioning.

Although both sensory sensitivity and avoiding reflect heightened detection of sensory input, individuals with sensory avoiding tend to exhibit stronger negative affective responses to such inputs. These aversive reactions may interfere with their social experiences. By contrast, individuals with sensory sensitivity—while demonstrating elevated perceptual acuity—often show comparatively attenuated negative reactivity, which may in turn limit its downstream impact on social functioning. In parallel, sensory seeking and registration share a profile of reduced perceptual responsiveness. However, children with registration difficulties often fail to detect salient social cues (e.g., eye gaze) and may show limited spontaneous monitoring of these signals. In comparison, sensory seekers actively pursue both sensory and social input, potentially compensating for diminished perceptual sensitivity. Consequently, sensory seeking is generally associated with a lesser degree of social impairment.

It is important to note that the cross-sectional design precludes causal inferences. Bidirectional or reverse pathways—such as social difficulties exacerbating sensory or executive challenges—are also plausible and should be explored in longitudinal studies.

### 4.4 Clinical implications

The relationship between sensory processing abnormalities and social impairments in children with ASD appears to be underpinned by deficits in higher-order cognitive processes, particularly in behavioral and emotional regulation. These findings highlight the importance of integrating executive function training into clinical interventions. For children with registration difficulties, interventions should target not only sensory processing but also behavioral regulation to improve social adaptive skills. For those with sensory avoidance, therapies should aim not only to modulate sensory input but also to enhance emotional and behavioral regulation capacities. Such integrated approaches may more effectively improve social outcomes in ASD.

### 4.5 Limitations

Several limitations should be considered. First, all constructs were assessed using parent-reported measures, which provide valuable ecological validity but may be subject to reporting biases. Future studies should incorporate objective measures, such as behavioral tasks and physiological assessments, to triangulate these findings. Second, although a priori power analysis indicated sufficient statistical power for detecting medium-to-large effects, the modest sample size may have limited our ability to identify smaller effects. Third, all participants had IQ scores above 70, which restricts the generalizability of the findings to individuals with co-occurring intellectual disability. Nevertheless, by focusing on children without intellectual impairments, this study allowed a clearer examination of the contributions of higher-order cognitive processes to sensory and social functioning. Fourth, the use of parent-reported measures with overlapping domains may partially explain the mediation effects observed. Future studies should include more distinct and objective measures to disentangle these constructs. Fifth, the absence of a typically developing control group limits our ability to determine whether the observed mediation patterns are specific to ASD or reflect broader developmental trends in this age range. Future studies should include matched control groups to clarify the specificity of these mechanisms to ASD.

## 5 Conclusions

This study demonstrates that executive function—particularly behavioral and emotional regulation—mediates the relationship between sensory processing abnormalities and social responsiveness in children with ASD. Specifically, behavioral regulation partially mediated the effect of sensory avoidance, while emotional regulation showed a full mediation effect in this relationship, though the cross-sectional design warrants caution in interpreting full mediation as causal. Both behavioral and emotional regulation partially mediated the effect of registration difficulties on social responsiveness. These results suggest that sensory processing deficits may exacerbate impairments in emotion and behavioral regulation, which in turn contribute to social challenges. This research provides a novel theoretical framework for understanding the sensory–executive–social interplay in ASD and supports the development of interventions targeting executive processes to improve social functioning. Future longitudinal and experimentally designed studies are needed to examine how these mechanisms unfold across development and in different contexts.

## Data Availability

The original contributions presented in the study are included in the article/supplementary material. Further inquiries can be directed to the corresponding author.
